# Supplementary material for: Evidence of High-Intensity Exercise on Lower Limb Functional Outcomes and Safety in Acute and Subacute Stroke Population: A Systematic Review
Source: Int J Environ Res Public Health. 2022 Dec 22;20(1):153. doi: 10.3390/ijerph20010153 (PMC9819111; doi:10.3390/ijerph20010153)
Supplement: Supplementary file 1 [file ijerph-20-00153-s001.zip › ijerph-2013314-supplementary.pdf]

Supplementary Table S1. Key terms and search strategies for databases

Search	Query
#1	Stroke[Mesh] OR "cerebrovascular accident"[tw] OR "cerebrovascular disorder"[tw] OR "ischemic stroke"[tw] OR “hemorrhagic stroke”[tw] OR CVA [tw] OR CVAs [tw] OR “cerebrovascular apoplexy”[tw] OR “apoplexy”[tw] OR "intracerebral hemorrhage" [tw] OR "cerebral hemorrhage" [tw] OR "cerebral infarction"[tw] OR "brain infarction"[tw] OR "brain ischemia"[tw] OR ((“stroke” [Mesh] OR “stroke” [tw]) AND (“cerebrum” [Mesh] OR “cerebrum” [tw] OR “cerebral” [tw] OR “brain” [Mesh] OR “brain” [tw]))
#2	"intensity training"[tw] OR "intensity exercise"[tw] OR "aerobic intensity"[tw] OR (intens*[tw] AND (exercise [mesh] OR training [tw] OR "physical activit*”[tw] OR “physical therapy”[tw] OR "physical education and training"[Mesh] OR sports[mesh] OR "exercise therapy"[tw] OR "motor activity"[Mesh])) OR “aerobic interval training”[tw] OR (“aerobic interval'”[tw] AND (exercise [Mesh] OR “training”[tw] OR “physical activit*”[tw] OR “physical therapy”[tw] OR “physical education and training”[Mesh] ORsports [Mesh] OR “exercise therapy”[tw] OR “motor activity”[Mesh]))
#3	"high intensity training"[tw] OR "high intensity exercise"[tw] OR "high intensity"[tw] OR "high-aerobic intensity" OR (“high intens*”[tw] AND (exercise [mesh] OR training [tw] OR "physical activit*”[tw] OR “physical therapy”[tw] OR "physical education and training"[Mesh] OR sports[mesh] OR "exercise therapy"[tw] OR "motor activity"[Mesh])) OR "continuous moderate exercise"[tw] OR ("continuous"[tw] AND "moderate"[tw] AND (exercise [mesh] OR training [tw] OR "physical activit*”[tw] OR “physical therapy”[tw] OR "physical education and training"[Mesh] OR sports[Mesh] OR "exercise therapy"[tw] OR "motor activity"[Mesh]))
#4	“acute” [tw] OR “subacute” [tw] OR “early” [tw]

#5	functional outcomes OR function OR gait OR walking
#6	#1 AND (#2 OR #3) AND #4 AND #5
